# Research progress of traditional Chinese medicine monomer in treating diabetic peripheral neuropathy: A review

**DOI:** 10.1097/MD.0000000000037767

**Published:** 2024-03-29

**Authors:** Shi Xiaoqin, Tian Yi, Liu Xiaoyu, Bu Ya, Shui Jingwen, Liping Yin

**Affiliations:** a Chengdu University of Traditional Chinese Medicine, Sichuan, China; b Affiliated Hospital of Chengdu University of Traditional Chinese Medicine, Sichuan, China.

**Keywords:** Chinese medicine, compound prescription, diabetic peripheral neuropathy, traditional Chinese medicine monomer

## Abstract

Diabetes peripheral neuropathy is one of the most common complications of diabetes. Early symptoms are insidious, while late symptoms mainly include numbness, pain, swelling, and loss of sensation in the limbs, which can lead to disability, foot ulcers, amputation, and so on. At present, the pathogenesis is also complex and diverse, and it is not yet clear. Western medicine treatment mainly focuses on controlling blood sugar and nourishing nerves, but the effect is not ideal. In recent years, it has been found that many drug monomers have shown good therapeutic and prognostic effects in the prevention and treatment of diabetes peripheral neuropathy, and related research has become a hot topic. To understand the specific mechanism of action of traditional Chinese medicine monomers in treatment, this article provides a review of their mechanism research and key roles. It mainly includes flavonoids, phenols, terpenes, saponins, alkaloids, polysaccharides, etc. By nuclear factor-κB (NF-κB), the signaling pathways of adenosine monophosphate-activated protein kinase (AMPK), Nrf2/ARE, SIRT1/p53, etc, can play a role in lowering blood sugar, antioxidant, anti-inflammatory, inhibiting cell apoptosis, and autophagy, promoting sciatic nerve regeneration, and have great potential in the prevention and treatment of this disease. A systematic summary of its related mechanisms of action was conducted, providing ideas for in-depth research and exploration of richer traditional Chinese medicine components, and also providing a relatively complete theoretical reference for clinical research on diabetes peripheral neuropathy treatment.

## 1. Introduction

Diabetes peripheral neuropathy (DPN) is a kind of diabetes neuropathy. Epidemiological studies have found that by 2030, the number of diabetes patients will reach 578 million, and after 15 years, the number of affected people will reach 700 million,^[1]^ and more than 50% of diabetes patients will develop into DPN. The most common type is distal symmetric polyneuropathy, which is mainly characterized by loss of sensation in the limbs. If not treated in time in the early stage, it is easy to face the risk of infection, or even amputation, which will seriously affect the quality of life of diabetes patients. At present, there are no specific effective drugs available. The existing drugs mainly focus on controlling blood sugar, nourishing nerves, controlling metabolic syndrome, and relieving pain.^[2]^ However, most treatments can only alleviate the symptoms of patients and fail to fundamentally solve the problem. Therefore, it is urgent to find safe and effective treatment drugs. Compared with Western medicine, traditional Chinese medicine has the characteristics of multiple targets, multiple components, and multiple pathways. In recent years, the government has increased its support for the traditional Chinese medicine industry, and more and more research has been conducted on the use of traditional Chinese medicine monomers to treat DPN. This article summarizes the basic and clinical research of common traditional Chinese medicine monomers for treating DPN, providing a reference for further exploring its pathogenesis and clinical medication.

## 2. The role of traditional Chinese medicine monomer in the treatment of DPN

### 2.1. Flavonoids

Modern studies have reported that flavonoids have anti-inflammatory, antioxidant, and blood sugar control effects. Puerarin is the main active extract of *Pueraria lobata* and an isoflavone compound. Xue et al^[3]^ found that puerarin protects tissue cells from damage by inhibiting cell apoptosis and oxidative stress, which is related to its inhibition of activation of apoptosis-related proteins (including caspase-3), downregulation of bcl-2, and upregulation of Bax. Zhao et al^[4]^ confirmed that quercetin can improve the mechanical threshold, sciatic nerve conduction velocity, and pathological changes of the sciatic nerve in diabetes rats, possibly by downregulating TLR4/MyD88/NF-κB signaling pathway to reduce the level of inflammatory factors in diabetes rats. Park et al^[5]^ found that baicalin inhibits excessive reactive oxygen species (ROS) and blocks hydrogen peroxide (H_2_O_2_) induced apoptosis and DNA damage in RT4-D6P2T Schwann cells, indicating that baicalin can protect Schwann cells from oxidative stress. Li et al^[6]^ found through animal experiments that luteolin can reduce the levels of ROS and malondialdehyde in the blood of mice to a certain extent. This experiment showed that luteolin can resist oxidation and alleviate peripheral nerve damage in DPN rats. In addition, it was found that luteolin upregulates the expression of Nrf2 and HO-1 (a phase II detoxifying enzyme) by activating the Nrf2 pathway. This series of antioxidant mechanisms can balance nerve damage caused by free radicals, thereby achieving a protective effect on nerves. Kishore et al^[7]^ found that kaempferol can correct hyperglycemia and partially reverse the pain response of diabetes rats by regulating oxidative and nitrous stress and reducing the formation of AGEs in diabetes rats. Naringenin is a natural flavonoid with neuroprotective activity. Naringenin is a natural flavonoid with neuroprotective activity. Semis et al^[8]^ have shown that naringenin can alleviate peripheral neuropathy caused by oxaliplatin, and its mechanism of action is related to improving SOD, CAT, Glutathione peroxidase (GPx), Nrf2, HO-1, TNF-α, etc. Another study suggests that naringin is expressed through AMPK-α And PKC-δ. The pathway activates cytokine signaling (SOCS)-3 in microglia to inhibit the expression of nitric oxide synthase (iNOS), cyclooxygenase-2 (COX-2), and pro-inflammatory cytokines.^[9]^

### 2.2. Polyphenols

Turmeric is the root of the herb ginger, and curcumin is a chemical extract of turmeric. Modern studies have confirmed that it can inhibit the expression levels of ROS, nitric oxide (NO), protein kinase C (PKC), tumor necrosis factor (TNF)-α, interleukin (IL)-1β, interleukin (IL)-6, NF-κB, etc, which indicates that it can play an antioxidant and anti-inflammatory role to protect peripheral nerves from damage.^[10–12]^ Curcumin inhibits astrocyte proliferation, microglial activation, and neuronal apoptosis by regulating Nrf2/HO-1 and NF-kB signaling pathways.^[13,14]^ Sang et al^[15,16]^ showed that it can also stimulate nerve growth factor (NGF) by regulating TrkA and PI3K/Akt signaling pathway, regulates autophagy-related cells, and promote myelin regeneration and axon regeneration, suggesting that curcumin can prevent Schwann cell apoptosis through autophagy, promote NGF, and promote myelination to accelerate the repair of rat sciatic nerve injury. Oxidative stress induced by a high-glucose environment is one of the causes of DPN. Zhang et al^[17]^ demonstrated that resveratrol stimulates the expression of Nrf2, inhibits the NF-KB pathway, and reduces the expression of neurofactors, including monocyte chemotactic protein-1 (MCP-1) and TNF-α and IL-1β, To protect peripheral nerves from apoptosis. Meng et al^[18]^ demonstrated that resveratrol can inhibit NF-κB activity, reducing IL-6 and TNF-α and the expression of inflammatory factors such as COX-2. An experimental study showed that resveratrol was administered to Wistar rats for 6 weeks after STZ-induced diabetes, and after 6 weeks resveratrol treatment significantly reduced MDA, XO, and NO production and increased glutathione levels compared to the control group. Resveratrol is an effective neuroprotective agent against diabetic oxidative damage.^[19]^ Studies have shown that resveratrol can inhibit the expression of TNF-α and IL-6 in the NF-κB signaling pathway and peripheral nerves, and activate the Nrf2/ARE signaling pathway to improve oxidative stress, increase sciatic nerve conduction velocity and intraneural blood flow in diabetic rats, and thus play a protective role in diabetic peripheral nerves.^[20]^ Research shows. Polydatin^[21]^ can improve mitochondrial function, reduce mitochondrial superoxide (mt SOX) by activating AMPK/PGC-1α pathway, and upregulate the expression of sirtuin (SIRT1), Nrf1, and m RNA. In terms of inflammation, polydatin has been shown to inhibit toll-like receptor-2 (TLR-2) and NF-κB p65 to reduce inflammatory cytokines.^[22]^ A large number of scientific studies have reported that magnolol has antioxidant, anti-inflammatory, analgesic, neuroprotective, and other biological activities.^[23]^ Yang et al^[24]^ showed that magnolol inhibited apoptosis by inhibiting PPARγ/NF-κB signaling pathway, regulating Bcl-2 family proteins mediated by PPARγ in dorsal root ganglion and sciatic nerve of DPN rats, and promoting axon growth of DRG neurons. The mitochondrial basal respiration rate and ATP production of DPN mice were increased, the levels of MMP, mitochondrial complex I and IV (COX) were upregulated, and the mitochondrial bioenergy levels of DRG neurons of DPN mice were restored.

### 2.3. Terpenoids

Zhu et al^[25]^ found that paeoniflorin interferes with SCs exosomes in a high-glucose environment, affecting the expression of proapoptotic protein in DRG neurons, increasing the expression of antiapoptotic protein, and reducing the apoptosis induced by endoplasmic reticulum stress to improve DPN. Yang et al^[26]^ confirmed that paeoniflorin can reduce demyelination of sciatic nerve tissue and improve mechanical pain threshold by upregulating Trx reductase 2 (Trx2), indicating that paeoniflorin can improve mitochondrial function in high-glucose environments, which may be one of its mechanisms of protecting DPN nerve tissue. Zhang et al^[27]^ studies have shown that tanshinone IIA can reduce pro-inflammatory factors (IL-1, TNF-α, IL-6) and increase interleukin-(IL-)10, so as to reduce the excessive inflammatory response and protect peripheral nerves. Xu et al^[28]^ demonstrated that andrographolide can accelerate the in vitro proliferation of RSC96 Schwann cells and maintain the phenotype of Schwann cells. Wang et al^[29]^ have shown that Androgynous lotus can relieve abnormal pain caused by nerve injury, which may be closely related to the secretion of inflammatory factors. In cell experiments, loganin^[30]^ has been confirmed to prevent RSC96 Schwann cell apoptosis by inhibiting ROS production, reducing the expression of NF-κB, P2 × 7 purinergic receptor (P2X7R), and thioredoxin-interacting protein (TXNIP), and inhibiting the activation of NOD-like receptor protein 3 (NLRP3) inflammatosome and the cleavage of gasdermin D (GSDMD), thus reducing peripheral nerve injury.

### 2.4. Saponins

Dioscin is a natural steroidal saponin, which plays an important role in the treatment of diabetes peripheral neuropathy. Research has found that diosgenin can improve NF-κB. Malondialdehyde (MDA), superoxide dismutase (SOD), TNF-α, IL-1β, thereby improving the antioxidant system.^[31]^ Diosgenin inhibits the expression of COX-2 and iNOS in the sciatic nerve, indicating that diosgenin plays a repairing role in inflammatory damage to the sciatic nerve.^[32]^ Research has shown that astragaloside IV can prevent mitochondrial dysfunction in central neurons by increasing SOD and glutathione (GSH), reducing MDA, inhibiting oxidative stress, and protecting peripheral nerves by regulating the SIRT1/p53 signaling pathway.^[33]^ In addition, astragaloside IV promotes glutathione activity and increases advanced glycation end products (AGEs), thereby delaying peripheral nerve damage in streptozotocin (STZ) treated rats.^[34]^ Yin et al^[35]^ established in vitro and in vivo models and found that astragaloside IV can inhibit the expression of the PI3K/Akt/mTOR signaling pathway, upregulates the expression of Beclin-1 and LC3 proteins, thereby reducing blood sugar in DPN rats and alleviating peripheral nerve damage. Ginsenoside is an extract of traditional Chinese medicine ginseng. Ginsenoside Rb1 can inhibit cell apoptosis and oxidative reactions in high-glucose environments. There are also studies indicating that the protective effect of ginsenosides on nerves may be related to mitochondrial dysfunction.^[36]^ Glycyrrhetinic acid is the most abundant component in licorice. Studies by Ciarlo et al^[37]^ have shown that ammonium glycyrrhetinic acid can inhibit SH-SY5Y cell apoptosis and mitochondrial dysfunction in high-glucose environments, and alleviate neuropathic hypersensitivity induced by STZ in DPN mice.

### 2.5. Alkaloid compounds

Berberine, also known as berberine, is the main active ingredient of Chinese herbal medicines such as *Coptis chinensis* and *Phellodendron chinensis.* As a natural activator of AMPK, it activates AMPK/PGC-1 by α pathway, upregulation of p-AMPK, and peroxisome proliferator-activated receptor gamma coactivator 1-alpha (PGC-1α), The expression of Nrf1 improves mitochondrial biogenic defects, reduces ROS generation, and promotes the antioxidant defense system of Nrf2 to function.^[38]^ Liu et al^[39]^ showed that berberine can alleviate neuropathic pain caused by DPN, which is related to its inhibition of the expression of pro-inflammatory cytokines and TNF-α, IL-6, and IL-1β and related to iNOS and COX-2. Agoons et al^[40]^ confirmed that local application of capsaicin can effectively alleviate pain caused by DPN in the second to 6th week after treatment, but this therapeutic effect will disappear after the 6th week. Some studies also suggest that the treatment of DPN with capsaicin is related to its concentration. High concentrations of capsaicin can inhibit the expression of the transient receptor potential vanilloid 1 (TRPV1), leading to long-term dysfunction of afferent nerve endings and achieving analgesic effects.^[41]^

### 2.6. Polysaccharides

Wang et al^[42]^ intervened in high glucose-induced SCs by using water extracts of Aconite. After 48 hours of treatment, they found that aconite polysaccharides significantly reduced intracellular ROS, mainly manifested in upregulation of SOD, catalase (CAT), and PGC-1α. Downregulation of nicotinamide adenine dinucleotide phosphate oxidase-1 protein level at protein level significantly increases AMPK activation. Preliminary inference suggests that aconite polysaccharides may regulate AMPK/PGC-1 by α The signaling pathway plays a protective role against high glucose-induced cell damage. Liu et al^[43]^ showed that Lycium polysaccharide can inhibit the mTOR/p70S6K signaling pathway, increase the expression of sciatic nerve-related proteins (LC3-II, Beclin-1) in DPN rats, and inhibit P62 protein levels. This indicates that the protective effect of Lycium polysaccharide on DPN may be achieved by inducing autophagy.

### 2.7. Others

Salvianolic acid is considered the most abundant compound, and modern pharmacological studies have confirmed that salvianolic acid A (Sal A) and salvianolic acid B (Sal B) have antioxidant and antiapoptotic effects. Xu et al^[44]^ found that Sal A can alleviate high glucose-induced damage by inhibiting 1,1-diphenyl-2-picrylhydrazine (DPPH) and reducing the levels of ROS, MDA, and oxidized glutathione (GSSG), as well as upregulating the expression of antioxidant enzyme mRNA, exhibiting strong antioxidant effects. Wang et al^[45]^ confirmed that Sal B inhibited the expression of JNK pathway, the levels of apoptosis and autophagy-related proteins such as poly-ADP-ribose polymerase (PARP), cleaved caspase-3, cleaved caspase-9, Beclin-3, LC3-A/B were down-regulated. Inhibit apoptosis and autophagy, and have a protective effect on SCs in high-glucose environment. *Achyranthes bidentata* polypeptide K (ABPPk) is a compound of *A. bidentata* polypeptide. ABPPk has been shown to inhibit Schwann cell apoptosis by regulating the PI3K/AKT and ERK1/2 signaling pathways.^[46]^ ABPPk is a light antioxidant active factor that needs to be further explored in the treatment of DPN and the prevention of neurological diseases. Melatonin is a hormone known to have the ability to resist oxidative stress. Tiong et al^[47]^ found that melatonin can change NF-κB, Bcl-2, Wnt, and mTOR signals through antioxidant capacity, thereby reducing apoptosis of Schwann cells.

## 3. Summary and outlook

As of now, the pathogenesis of DPN has not been fully understood. Traditional Chinese medicine believes that its pathogenesis is related to dysfunction of the organs, phlegm stasis, and imbalance of qi, blood, yin, and yang. However, modern research suggests that it is caused by advanced glycation end products, oxidative stress, and mitochondrial disorders, activation of polyol pathways, nitration reactions, and endoplasmic reticulum stress. In recent years, scholars at home and abroad have demonstrated the mechanism of traditional Chinese medicine formulas and extracts in treating DPN, A large amount of basic research and clinical trials have been conducted, providing a significant theoretical basis for the prevention and treatment of DPN. The main signaling pathways involved in the treatment of DPN with traditional Chinese medicine formulas and monomers are NF-κB. The mechanisms involved in AMPK, Nrf2/ARE, JNK, miR-211, SIRT1/p53, etc, mainly include: controlling blood sugar, antioxidant, anti-inflammatory, inhibiting cell apoptosis, inhibiting autophagy, improving metabolic syndrome, etc. Each target is interconnected and jointly affects, exerting its protective effect on neuropathy.

Although many studies have not explicitly expressed the side effects of traditional Chinese medicine, the flexible application of traditional Chinese medicine has added challenges to the study of its mechanism for treating DPN. To better avoid the side effects of traditional Chinese medicine, research on the treatment of DPN with traditional Chinese medicine monomers is receiving increasing attention. This article reviews the protective effects of monomers, such as puerarin, baicalin, luteolin, paeoniflorin, and astragalus polysaccharides on DPN. The specific mechanism is detailed in Table 1. To translate the therapeutic potential of DPN into reality, it is necessary to increase experimental research on single traditional Chinese medicine or its monomers in the future.

**Table 1:** Traditional Chinese medicine monomer therapy for diabetes peripheral neuropathy.

Traditional Chinese medicine monomer	Model	Intervention methods	Pharmacology	Index	References
Puerarin	Schwann cells	Cell culture	Inhibit apoptosis and oxidative stress	bax↑; bcl-2, ROS↓, Inhibition of PARP and caspase-3 activation	^[3]^
Quercetin	DPN rat	Gavage	Reduce levels of inflammatory factors	TLR4, myd 88, NF-κB, TNF-α, IL-1β↓	^[4]^
Baicalin	RT4-D6P2T cells	Cell culture	Antioxidation and neuroprotection	ROS, Bax/Bcl-2↓	^[5]^
Luteolin	Diabetic rats	Gavage	Antioxidation and neuroprotection	NRF 2, HO-1↑; ROS, MDA↓	^[6]^
Kaempferol	Wistar rats	intraperitoneal injection	Regulation of oxidative and nitrite stress	AGEs↓	^[7]^
Naringenin	Rats	Gavage	Reduced oxaliplatin induced oxidative stress, inflammation, and apoptosis in sciatic nerve tissue	SOD, CAT, GPx, Nrf2, HO-1, TNF-α↑	^[8]^
	The murine microglial cell BV-2 and N2a cells	Cell culture	Inhibit neuroinflammation	iNOS, COX-2↓; Increases PKCδ Activation	^[9]^
Curcumin	SD rats	Gavage	It inhibited spinal cord central neuropathy, glial cell activation and neuronal apoptosis in diabetic patients.	NF-kB↓	^[14]^
	PC-12 cells	Cell culture	Stimulating the release of NGF can protect the damaged neurons	TrkA, Akt, NGF↑	^[15]^
Resveratrol	DPN rat	Gavage	Anti-inflammatory	Nrf2↑; MCP-1, TNF-α, IL-1 β↓;inhibiting the NF-KB pathway	^[17]^
	Wistar rats	Intraperitoneal injection	Resist oxidative damage	GSH↑; MDA, XO, NO↓	^[19]^
Polydatin	SD rat	Gavage	Improving mitochondrial function	SIRT1, Nrf2, TFAM, m RNA↑; mt SOX↓	^[21]^
Magnolol	Diabetic rat	Gavage	Promoting axonal growth of DRG neurons	ATP, MMP, COX↑	^[24]^
Paeoniflorin	Schwann cells	Cell culture	Reducing endoplasmic reticulum stress-induced cell apoptosis	IRE1α↓	^[25]^
	RSC96 cells	Cell culture	Upregulates mitochondrial thioredoxin of Schwann Cells	Trx2↑	^[26]^
Tanshinone IIA	Diabetic rat	Gavage	Anti-inflammatory	IL-10↑; IL-1β, IL-6, TNF-α↓	^[27]^
loganin	Schwann cells	Cell culture	Antioxidation and anti-inflammatory properties	ROS, NF-κB, P2X7R, TXNIP, NLRP3↓; Inhibition of GSDMD cleavage	^[30]^
Astragaloside	Schwann cells	Cell culture	Enhanced autophagy	beclin-1, LC3↑	^[35]^
Berberine	Diabetic rat	Gavage	Inhibiting the expression of pro-inflammatory factors	TNF-α, IL-6, IL-1β, iNOS, COX-2↓	^[39]^
Aconite polysaccharide	Schwann cells	Cell culture	Antioxidative stress, reduce cell apoptosis	CAT, SOD, PGC-1α↑; ROS↓	^[42]^
Lycium polysaccharide	Diabetic rat	Gavage	Enhancing cellular autophagy	LC3-II, Beclin-1↑; P62↓	^[43]^
Salvianolic acid A	Schwann cells	Cell culture	Reduce the production of ROS and alleviate neuroinflammation	ROS, GSSG, MDA, DPPH↓	^[44]^
Salvianolic acid B	Schwann cells	Cell culture	Inhibiting excessive autophagy	PARP, cleaved caspase-3, cleaved caspase-9, Beclin-3, LC3A/B↓	^[45]^

↑Stimulate and enhance; ↓reduce, inhibit.

AMPK = adenosine monophosphate activated protein kinase, BCL-2 = Beclin-2, CAT = catalase, COX-2 = cyclooxygenase-2, DPN = diabetes peripheral neuropathy, DRG = dorsal root ganglion, GFAP = glia fibrillary acidic protein, GPx = glutathione peroxidase, GRP78 = The glucose-regulated protein 78, GSDMD = gasdermin D, GSH = glutathione, GSSG = oxidized glutathione, HO-1 = heme oxygenase-1, Iba1 = ionized calcium-binding adaptor molecule 1, IL-6 = interleukin-6, IL-10 = interleukin-10, IRE1α = inositol-requiring kinase 1, MCP-1 = monocyte chemotactic protein-1, MDA = malondialdehyde, mt SOX = mitochondrial superoxide, mTOR = mammalian target of rapamycin, NeuN = neuronal nuclear protein, NF-κB = nuclear factor kappa B, NLRP3 = NOD-like receptor protein 3, NO = nitric oxide, Nrf2 = nuclear factor E2-related factor 2, P2X7R = P2 × 7 purinergic receptor, PARP = poly-ADP-ribose polymerase, PGC-1α = peroxisome proliferator-activated receptor gamma coactivator 1-alpha, Prx3 = peroxiredoxin 3, ROS = reactive oxygen species, SD rat = Sprague–Dawley rat, SIRT1 = sirtuin, SOD = superoxide dismutase, TFAM = mitochondrial transcription factor A, TLR-2 = toll-like receptor-2, TNF-α = tumor necrosis factor, TrxR2 = Trx reductase 2, Trx2 = thioredoxin, TXNIP = thioredoxin-interacting protein, XO = xanthine oxidase.

## 4. Conclusions

In summary, by reviewing the research on the treatment of DPN with traditional Chinese medicine monomers, the key role of traditional Chinese medicine treatment has been clarified, providing new ideas for later clinical research and treatment. However, due to the large number of traditional Chinese medicine monomers, research on their treatment of DPN in various aspects is not thorough, resulting in some problems in their current research: the mechanism of traditional Chinese medicine treatment of DPN has not been integrated with traditional Chinese medicine theory; at present, traditional Chinese medicine monomers are widely used and mainly focused on animal experiments. However, the activation mechanisms are complex and diverse, and further cellular research is needed to confirm the exact target of action; the usage of traditional Chinese medicine in clinical practice is not clear, so its therapeutic effect is difficult to confirm. In the future, the efficacy, adverse reactions, and mechanism of action of traditional Chinese medicines, monomer drugs, and compounds can be summarized. On the one hand, the specific principle of their action on diseases can be more clearly understood. On the other hand, using methods such as component omics, network pharmacology and molecular docking, bioinformatics, and high-throughput mass spectrometry, we explore the specific active ingredients of traditional Chinese medicine formulas and monomeric drugs entering the human body or other experimental animals. After specific research, we can seek benefits and avoid harm, and dismantle and verify the original classic formulas, which may make traditional Chinese medicine monomers more scientifically and effectively applied in clinical practice.

## Author contributions

**Resources:** Xiaoqin Shi, Jingwen Shui, Xiaoyu Liu, Ya Bu.

**Writing—original draft:** Xiaoqin Shi.

**Writing—review & editing:** Xiaoqin Shi.

**Data curation:** Yi Tian.

**Methodology:** Liping Yin.

## References

[1] Saeedi P Petersohn I Salpea P.; IDF Diabetes Atlas Committee. Global and regional diabetes prevalence estimates for 2019 and projections for 2030 and 2045: results from the International Diabetes Federation Diabetes Atlas, 9th edition. Diabetes Res Clin Pract. 2019;157:107843. 31518657 10.1016/j.diabres.2019.107843

[2] Elafros MA Andersen H Bennett DL. Towards prevention of diabetic peripheral neuropathy: clinical presentation, pathogenesis, and new treatments. Lancet Neurol. 2022;21:922–36. 36115364 10.1016/S1474-4422(22)00188-0 PMC10112836

[3] Xue B Wang L Zhang Z. Puerarin may protect against Schwann cell damage induced by glucose fluctuation. J Nat Med. 2017;71:472–81. 28181078 10.1007/s11418-016-1067-0

[4] Zhao B Zhang Q Liang X. Quercetin reduces inflammation in a rat model of diabetic peripheral neuropathy by regulating the TLR4/MyD88/NF-κB signalling pathway. Eur J Pharmacol. 2021;912:174607. 34743981 10.1016/j.ejphar.2021.174607

[5] Park C Choi EO Kim GY. Protective effect of baicalein on oxidative stress-induced DNA damage and apoptosis in RT4-D6P2T Schwann cells. Int J Med Sci. 2019;16:8–16. 30662323 10.7150/ijms.29692 PMC6332490

[6] Li M Li Q Zhao Q. Luteolin improves the impaired nerve functions in diabetic neuropathy: behavioral and biochemical evidences. Int J Clin Exp Pathol. 2015;8:10112–20. 26617718 PMC4637533

[7] Kishore L Kaur N Singh R. Effect of Kaempferol isolated from seeds of *Eruca sativa* on changes of pain sensitivity in streptozotocin-induced diabetic neuropathy. Inflammopharmacology. 2018;26:993–1003. 29159712 10.1007/s10787-017-0416-2

[8] Semis HS Kandemir FM Caglayan C. Protective effect of naringin against oxaliplatin-induced peripheral neuropathy in rats: a behavioral and molecular study. J Biochem Mol Toxicol. 2022;36:e23121. 35670529 10.1002/jbt.23121

[9] Wu LH Lin C Lin HY. Naringenin suppresses neuroinflammatory responses through inducing suppressor of cytokine signaling 3 expression. Mol Neurobiol. 2016;53:1080–91. 25579382 10.1007/s12035-014-9042-9

[10] Caillaud M Thompson D Toma W. Formulated curcumin prevents paclitaxel-induced peripheral neuropathy through reduction in neuroinflammation by modulation of α7 nicotinic acetylcholine receptors. Pharmaceutics. 2022;14:1296. 35745868 10.3390/pharmaceutics14061296 PMC9227889

[11] Zeng Y Luo Y Wang L. Therapeutic effect of curcumin on metabolic diseases: evidence from clinical studies. Int J Mol Sci. 2023;24:3323. 36834734 10.3390/ijms24043323 PMC9959718

[12] Basu P Maier C Basu A. Effects of curcumin and its different formulations in preclinical and clinical studies of peripheral neuropathic and postoperative pain: a comprehensive review. Int J Mol Sci. 2021;22:4666. 33925121 10.3390/ijms22094666 PMC8125634

[13] Caillaud M Chantemargue B Richard L. Local low dose curcumin treatment improves functional recovery and remyelination in a rat model of sciatic nerve crush through inhibition of oxidative stress. Neuropharmacology. 2018;139:98–116. 30018000 10.1016/j.neuropharm.2018.07.001

[14] Elsayed HRH Rabei MR Elshaer MMA. Suppression of neuronal apoptosis and glial activation with modulation of Nrf2/HO-1 and NF-kB signaling by curcumin in streptozotocin-induced diabetic spinal cord central neuropathy. Front Neuroanat. 2023;17:1094301. 36968023 10.3389/fnana.2023.1094301 PMC10035597

[15] Sang Q Sun D Chen Z. NGF and PI3K/Akt signaling participate in the ventral motor neuronal protection of curcumin in sciatic nerve injury rat models. Biomed Pharmacother. 2018;103:1146–53. 29715758 10.1016/j.biopha.2018.04.116

[16] Zhao Z Li X Li Q. Curcumin accelerates the repair of sciatic nerve injury in rats through reducing Schwann cells apoptosis and promoting myelinization. Biomed Pharmacother. 2017;92:1103–10. 28622711 10.1016/j.biopha.2017.05.099

[17] Zhang W Yu H Lin Q. Anti-inflammatory effect of resveratrol attenuates the severity of diabetic neuropathy by activating the Nrf2 pathway. Aging. 2021;13:10659–71. 33770763 10.18632/aging.202830 PMC8064179

[18] Meng T Xiao D Muhammed A. Anti-inflammatory action and mechanisms of resveratrol. Molecules. 2021;26:229. 33466247 10.3390/molecules26010229 PMC7796143

[19] Ates O Cayli SR Yucel N. Central nervous system protection by resveratrol in streptozotocin-induced diabetic rats. J Clin Neurosci. 2007;14:256–60. 17258134 10.1016/j.jocn.2005.12.010

[20] Ahmad I Hoda M. Attenuation of diabetic retinopathy and neuropathy by resveratrol: review on its molecular mechanisms of action. Life Sci. 2020;245:117350. 31982401 10.1016/j.lfs.2020.117350

[21] Bheereddy P Yerra VG Kalvala AK. SIRT1 activation by polydatin alleviates oxidative damage and elevates mitochondrial biogenesis in experimental diabetic neuropathy. Cell Mol Neurobiol. 2021;41:1563–77. 32683581 10.1007/s10571-020-00923-1 PMC11448605

[22] Lv R Du L Zhang L. Polydatin attenuates spinal cord injury in rats by inhibiting oxidative stress and microglia apoptosis via Nrf2/HO-1 pathway. Life Sci. 2019;217:119–27. 30481506 10.1016/j.lfs.2018.11.053

[23] Szałabska-Rąpała K Borymska W Kaczmarczyk-Sedlak I. Effectiveness of magnolol, a lignan from Magnolia Bark, in diabetes, its complications and comorbidities: a review. Int J Mol Sci. 2021;22:10050. 34576213 10.3390/ijms221810050 PMC8467064

[24] Yang J Wei Y Zhao T. Magnolol effectively ameliorates diabetic peripheral neuropathy in mice. Phytomedicine. 2022;107:154434. 36122436 10.1016/j.phymed.2022.154434

[25] Zhu Y Han S Li X. Paeoniflorin effect of Schwann cell-derived exosomes ameliorates dorsal root ganglion neurons apoptosis through IRE1α pathway. Evid Based Complement Alternat Med. 2021;2021:6079305. 34616478 10.1155/2021/6079305 PMC8490051

[26] Yang X Li X Zhu Y. Paeoniflorin upregulates mitochondrial thioredoxin of Schwann cells to improve diabetic peripheral neuropathy indicated by 4D label-free quantitative proteomics. Oxid Med Cell Longev. 2022;2022:4775645. 35340203 10.1155/2022/4775645 PMC8956397

[27] Zhang B Yu Y Aori G. Tanshinone IIA attenuates diabetic peripheral neuropathic pain in experimental rats via inhibiting inflammation. Evid Based Complement Alternat Med. 2018;2018:2789847. 29713362 10.1155/2018/2789847 PMC5866893

[28] Xu F Wu H Zhang K. Pro-neurogenic effects of andrographolide on RSC96 Schwann cells in vitro. Mol Med Rep. 2016;14:3573–80. 27599453 10.3892/mmr.2016.5717 PMC5042728

[29] Wang HC Tsay HS Shih HN. Andrographolide relieved pathological pain generated by spared nerve injury model in mice. Pharm Biol. 2018;56:124–31. 29385888 10.1080/13880209.2018.1426614 PMC6130553

[30] Cheng YC Chu LW Chen JY. Loganin attenuates high glucose-induced Schwann cells pyroptosis by inhibiting ROS generation and NLRP3 inflammasome activation. Cells. 2020;9:1948. 32842536 10.3390/cells9091948 PMC7564733

[31] Kiasalari Z Rahmani T Mahmoudi N. Diosgenin ameliorates development of neuropathic pain in diabetic rats: involvement of oxidative stress and inflammation. Biomed Pharmacother. 2017;86:654–61. 28033582 10.1016/j.biopha.2016.12.068

[32] Leng J Li X Tian H. Neuroprotective effect of diosgenin in a mouse model of diabetic peripheral neuropathy involves the Nrf2/HO-1 pathway. BMC Complement Med Ther. 2020;20:126. 32336289 10.1186/s12906-020-02930-7 PMC7184706

[33] Ben Y Hao J Zhang Z. Astragaloside IV inhibits mitochondrial-dependent apoptosis of the dorsal root ganglion in diabetic peripheral neuropathy rats through modulation of the SIRT1/p53 signaling pathway. Diabetes Metab Syndr Obes. 2021;14:1647–61. 33883914 10.2147/DMSO.S301068 PMC8055373

[34] Li L Zhang Y Luo Y. The molecular basis of the anti-inflammatory property of astragaloside IV for the treatment of diabetes and its complications. Drug Des Devel Ther. 2023;17:771–90. 10.2147/DDDT.S399423 PMC10013573 36925998

[35] Yin Y Qu H Yang Q. Astragaloside IV alleviates Schwann cell injury in diabetic peripheral neuropathy by regulating microRNA-155-mediated autophagy. Phytomedicine. 2021;92:153749. 34601220 10.1016/j.phymed.2021.153749

[36] Xue B Sun L Li X. Ginsenoside Rb1 relieves glucose fluctuation-induced oxidative stress and apoptosis in Schwann cells. Neural Regen Res. 2012;7:2340–6. 25538758 10.3969/j.issn.1673-5374.2012.30.003 PMC4268738

[37] Ciarlo L Marzoli F Minosi P. Ammonium glycyrrhizinate prevents apoptosis and mitochondrial dysfunction induced by high glucose in SH-SY5Y cell line and counteracts neuropathic pain in streptozotocin-induced diabetic mice. Biomedicines. 2021;9:608. 34073550 10.3390/biomedicines9060608 PMC8227813

[38] Yerra VG Kalvala AK Sherkhane B. Adenosine monophosphate-activated protein kinase modulation by berberine attenuates mitochondrial deficits and redox imbalance in experimental diabetic neuropathy. Neuropharmacology. 2018;131:256–70. 29273519 10.1016/j.neuropharm.2017.12.029

[39] Liu M Gao L Zhang N. Berberine reduces neuroglia activation and inflammation in streptozotocin-induced diabetic mice. Int J Immunopathol Pharmacol. 2019;33:2058738419866379. 31337260 10.1177/2058738419866379 PMC6657114

[40] Agoons BB Dehayem Yefou M Katte JC. Effect of topical capsaicin on painful sensory peripheral neuropathy in patients with type 2 diabetes: a double-blind placebo-controlled randomised clinical trial. Cureus. 2020;12:e11147. 33251057 10.7759/cureus.11147 PMC7685815

[41] Arora V Campbell JN Chung MK. Fight fire with fire: neurobiology of capsaicin-induced analgesia for chronic pain. Pharmacol Ther. 2021;220:107743. 33181192 10.1016/j.pharmthera.2020.107743 PMC7969397

[42] Wang BB Wang JL Yuan J. Sugar composition analysis of Fuzi polysaccharides by HPLC-MSn and their protective effects on Schwann cells exposed to high glucose. Molecules. 2016;21:1496. 27834877 10.3390/molecules21111496 PMC6273632

[43] Liu SY Chen L Li XC. *Lycium barbarum* polysaccharide protects diabetic peripheral neuropathy by enhancing autophagy via mTOR/p70S6K inhibition in Streptozotocin-induced diabetic rats. J Chem Neuroanat. 2018;89:37–42. 29294366 10.1016/j.jchemneu.2017.12.011

[44] Xu C Hou B He P. Neuroprotective effect of salvianolic acid A against diabetic peripheral neuropathy through modulation of Nrf2. Oxid Med Cell Longev. 2020;2020:6431459. 32184918 10.1155/2020/6431459 PMC7063195

[45] Wang QQ Zhai C Wahafu A. Salvianolic acid B inhibits the development of diabetic peripheral neuropathy by suppressing autophagy and apoptosis. J Pharm Pharmacol. 2019;71:417–28. 30537209 10.1111/jphp.13044

[46] Li M Zhu Y Peng W. *Achyranthes bidentata* polypeptide protects Schwann cells from apoptosis in hydrogen peroxide-induced oxidative stress. Front Neurosci. 2018;12:868. 30555292 10.3389/fnins.2018.00868 PMC6284036

[47] Tiong YL Ng KY Koh RY. Melatonin prevents oxidative stress-induced mitochondrial dysfunction and apoptosis in high glucose-treated Schwann cells via upregulation of Bcl2, NF-κB, mTOR, Wnt signalling pathways. Antioxidants (Basel). 2019;8:198. 31247931 10.3390/antiox8070198 PMC6680940

